# Variation in susceptibility of eight insecticides in the brown planthopper *Nilaparvata lugens* in three regions of Vietnam 2015-2017

**DOI:** 10.1371/journal.pone.0204962

**Published:** 2018-10-05

**Authors:** Dao Bach Khoa, Bui Xuan Thang, Nguyen Van Liem, Niels Holst, Michael Kristensen

**Affiliations:** 1 Plant Protection Research Institute, Duc Thang, Bac Tu Liem, Hanoi, Vietnam; 2 Department of Agroecology, Aarhus University, Slagelse, Denmark; University of Crete, GREECE

## Abstract

The brown planthopper (BPH), *Nilaparvata lugens*, is a serious threat to rice production in Vietnam and insecticides are widely used for its control. Migration of the BPH have one of its roots in tropical Vietnam in the Mekong River Delta and the insecticide resistance status of BPH populations from Vietnam is thus important for East Asia. In the present investigation, we evaluate the susceptibility of BPH populations from nine provinces from the Red River Delta, the Central Coastal region and the Mekong River Delta of eight insecticides during 2015–17. BPH field populations of Vietnam have developed a low to moderate level of resistance to the neonicotinoids dinotefuran, nitenpyram and imidacloprid, the pyrethroid etofenprox, the anticholinesterase fenobucarb, as well as fipronil and pymetrozine, and the growth regulator buprofezin. There was a correlation of in toxicology of fipronil, dinotefuran, etofenprox, buprofezin, which represents four different modes of action. The neonicotinoid nitenpyram, pymetrozine and fenobucarb did not show correlation in toxicology to any of the investigated insecticides. For most insecticides, a gradient of susceptibility was established from the Red River Delta in the north, through the Central Coastal region and to the Mekong River Delta in the south of Vietnam. The most susceptible populations were from the north. Insecticide resistance of the BPH populations in Vietnam is not at an alarming level and they are not the direct origin of high insecticide resistance found in East Asia. The cross-resistance pattern of BPH populations in Vietnam, where insecticides with different modes of action correlated, indicate that insecticides should be used with caution. There could be a buildup of a general metabolic resistance, which alone or in combination with the emergence of target-site resistance mutations will cause control problems. The results will be beneficial for development of resistance management strategies to prevent and delay development of insecticide resistance in BPH not only for Vietnam, but also for more northern Asian regions due the migration of BPH from tropical Vietnam.

## Introduction

Rice, *Oryza sativa* L. (Graminales: Poaceae), is one of the important stable foods feeding nearly half of the world population. The brown planthopper (BPH), *Nilaparvata lugens* Stål, (Hemiptera: Delphacidae), is an important pest insect in most rice growing regions, especially in Asia. They damage rice directly through feeding causing hopper burns and by transmission of rice virus diseases such as grass stunt, ragged stunt and wilted stunt virus, respectively, e.g. causing yield loss of 2.7 million t during 2005–2007 in China [1–4].

Vietnam is one of the major rice-exporting countries and rice-production covers about 7.75 million ha with a yield of 43.3 mill t per year [5]. In Vietnam, BPH occurs in most of the rice-growing seasons and all regions. BPH caused serious hopper burns in Vietnam in 1978, 1991, 1992, 2006 and 2007 with losses of 700,000 t and 1 mill t in 2006 and 2007, respectively [4, 6].

Due to diverse topography and tropical monsoon climate, rice is grown in three main regions in different seasons in Vietnam. In northern Vietnam, the Red River Delta has two main rice-growing seasons, winter-spring (Feb-May) and summer-autumn (Jun-Oct). The Central Coast region has two rice-growing seasons, winter-spring (Jan-Apr) and summer-autumn (May-Nov). In south Vietnam the rice-growing seasons constantly varies based on the weather condition, floods and the dike systems, but mostly the Mekong River Delta has three rice-growing seasons, winter-spring (Dec-Mar), summer-autumn (Apr-Jul) and autumn-winter (Aug-Nov).

The BPH is a long-distance migratory pest. At rice blooming stage, a long-winged, migratory form develops and mass migration follows. In East Asia, the migration of the BPH has root in the Mekong Delta in tropical Vietnam and in the Philippines. Migration of BPH from the Vietnamese root goes northwards through Central Vietnam and maybe also from neighbouring Laos to the Red River Delta in northern Vietnam, depending on weather conditions, especially the wind. This completes the ‘first step migration’ [7]. This BPH population overwinters in the Red River Delta on rice and continues to migrate to southern China in the early summer [8], where the BPH pest population builds up on the first Chinese rice crop. This then initiates the ‘second step migration’ moving BPH populations to the Yangtze River Delta, the Korean Peninsula and Japan [8, 9]. As the BPH moves northwards through a range of agro-ecological zones, a selection of genetic traits can be expected. This selection is influenced by the range of rice cultivars grown with varying BPH resistance phenotypes [10, 11] and the control methods practiced, in the regions passed along the migration route. Exemplified by the 2005–2007 outbreak, where initially BPH resistance to imidacloprid occurred suddenly at a high frequency, all the way from Vietnam to Japan, and insecticide usage increased by 10,000 t per year in Vietnam in 2006 and 2007 compared to 2005 [12]. A better understanding of BPH population properties of the emigration regions are needed, especially for insecticide resistance risk assessment and better pest management practices.

The control of BPH worldwide is relaying on chemical insecticides including organophosphates, organochlorines, carbamates, pyrethroids, neonicotinoids as well as newer chemicals. Misuse and overuse of chemical insecticides have caused a series of disadvantages, which involved ecological and social problems [13]. The BPH has high potential for insecticide resistance development due to its distinct biological and behavioural characteristics, such as short development time, high fecundity and dispersal capacity. BPH have developed resistance against many insecticides with different modes of action [14–23].

In the last decades rice production in Vietnam is intensifying and increasing the use of agrochemicals e.g. insecticides. Monitoring and understanding of insecticide resistance is essential for successful pest management and BPH is not an exception. In this study we investigate insecticide resistance of the three rice growing regions of Vietnam, where the insecticide resistance ration is described for three populations in each region during 2015, 2016 and 2017. Based on the frequency of insecticide usage, which is more frequent in the south a gradient of susceptibility is hypothesized to be established from north to south Vietnam. The most susceptible populations are expected in the north. Due to the intensive use of insecticides the susceptibility is hypothesized to decrease from spring to autumn and from 2015 to 2017. We additionally hypothesize that the selection process will follow a pattern when following the correlation of LC_50_ and the slope of the dose-response regression line: i) an initial phase where the susceptible population will be characterized by a low LC_50_ and a steep slope of the regression line (homogenous susceptible population, but with some natural variation), ii) a second phase where selection with an insecticide will initially decrease the slope substantially and increase the LC_50_ slightly (heterogenous population, mixture of populations with different tolerance, iii) in the late stages of the selection of a resistance LC_50_ will increase significantly and a steep slope of the regression line will appear again (homogenous resistant population, with limited variation).

## Materials and methods

### Insects

The susceptible BPH population has been kept at the Plant Protection Research Institute in Hanoi since 1998. Nine populations of BPH were collected in rice paddy in the early winter-spring and summer-autumn rice-growing seasons fields in the Red River Delta provinces 1) VinhPhuc (21.22N, 105.53E), 2) HaiPhong (20.72N, 106.68E), 3) NamDinh (20.32N, 106.11E), in the central coast provinces 4) NgheAn (19.11N, 105.60E), 5) ThuaThien-Hue (16.47N, 107.53E), 6) PhuYen (13.09N, 109.27E) and in the Mekong Delta provinces 7) LongAn (10.75N, 105.81E), 8) AnGiang (10.61N, 105.09E), 9) SocTrang (9.60N, 105.77E) from 2015–2017 (https://www.google.com/maps/d/viewer?mid=1__q8b5g8g0JBbGV2GmAfPj9W5kt09Sgb&ll=15.867490234396235%2C103.93212317084965&z=5). Adults and nymphs of the first generation, 15–45 days after sowing, were collected in paddy fields. The brown plant hoppers were collected on private land with consent of the owner to the Plant Protection Research Institute, Pesticide, Weed and Environment Department, Hanoi, Vietnam. The field collection did not involve endangered or protected species.

The collected populations were reared on rice seedling (NT1 variety) in the laboratory with controlled-condition, temperature 37±1°C and 70–80% relative humidity with a 16-8h (L/D) photoperiod. The third nymph instars of the second laboratory generation were used for bioassay.

### Insecticides

Technical standard insecticides used for bioassay were technical grade pesticides. Dinotefuran (95%) and fipronil (95%) were supplied by SinoBio Chemistry, Dalian, China. Buprofezin (97%), fenobucarb (98%), pymetrozine (95%) were supplied by Shenzhen Golden Union Agrochemical, Shenzhen City, China. Etofenprox (95%), imidacloprid (95%), nitenpyram (95%) were supplied by Shanghai Mingdou Chemical, Shanghai, China. Stock solutions of the insecticides were made using technical grade acetone, then a gradient of concentrations was made using distilled water containing 0.1% Triton X-100.

### Bioassay

Susceptibility to insecticides was analyzed by a slightly modified from the IRAC Susceptibility Test #05 [24]. About ten rice seeds were sow in cups that contain alluvial soil with supplying NPK nutrient. When seedling rice had four leaves they were used for bioassay. Agar powder was diluted according to manufacturer’s instruction and allowed to cool to 37°C and then was poured on the rice seedling cups to cover the soil surface. Rice seedlings were dipped into six dose of insecticide solution for 30 s and kept at room temperature until the seedling dried (> 15 min). Ten third instar nymphs were transferred onto the rice seedling and the cup was covered with a plastic tube with a plastic mesh above. A test was replicated three times. Alive and death BPH’s was counted and recorded.

### Statistical analysis

A dose-response curve was estimated for every combination of year (3 years), locality (9 localities; 3 localities in each of 3 regions) and season (2 seasons; winter-spring and summer-autumn). This yielded a total of 54 dose-response curves for each of the 8 insecticides. However, this number was reduced to 53 as the data from PhuYen in spring 2016 were discarded; they showed an aberrant, low susceptibility to all insecticides and were considered as a technical outlier.

Dose-response curves were estimated by regression for each insecticide separately using the R drc package [25]. This package does now allow the specification of a statistical design, so each singular regression was given a unique key to identify it by year, region, locality and season; the 3 replicates for each such combination were pooled. This allowed pairwise comparisons of the 53 regression curves, or fewer, as the model was reduced. Model reduction was carried out by constructing alternative keys, leaving out one or more of year, region, locality and season from the key. Alternative models were tested used the anova.drc function, choosing the simpler model if P>0.05. Finally, levels of year and levels of region were merged, if the pairwise comparisons from drc allowed (P>0.05). However, only levels adjacent in time (2015–16 or 2016–17) or space (north-central or south-central) were merged. Differences in the final, reduced models were taken as statistically significant at P<0.05 by way of the compParm function, using ratio comparison for LC_50_ and difference comparison for slope. A dose-response curve was estimated separately for the susceptible laboratory strain for each insecticide.

Pearson correlation coefficients between LC_50_-values were computed for all pair-wise comparisons (n = 28) of insecticides using the R package "psych". Correlations were tested for significance adjusting the overall alpha = 0.05 for multiple comparisons [26].

## Results

**Fig 1:**
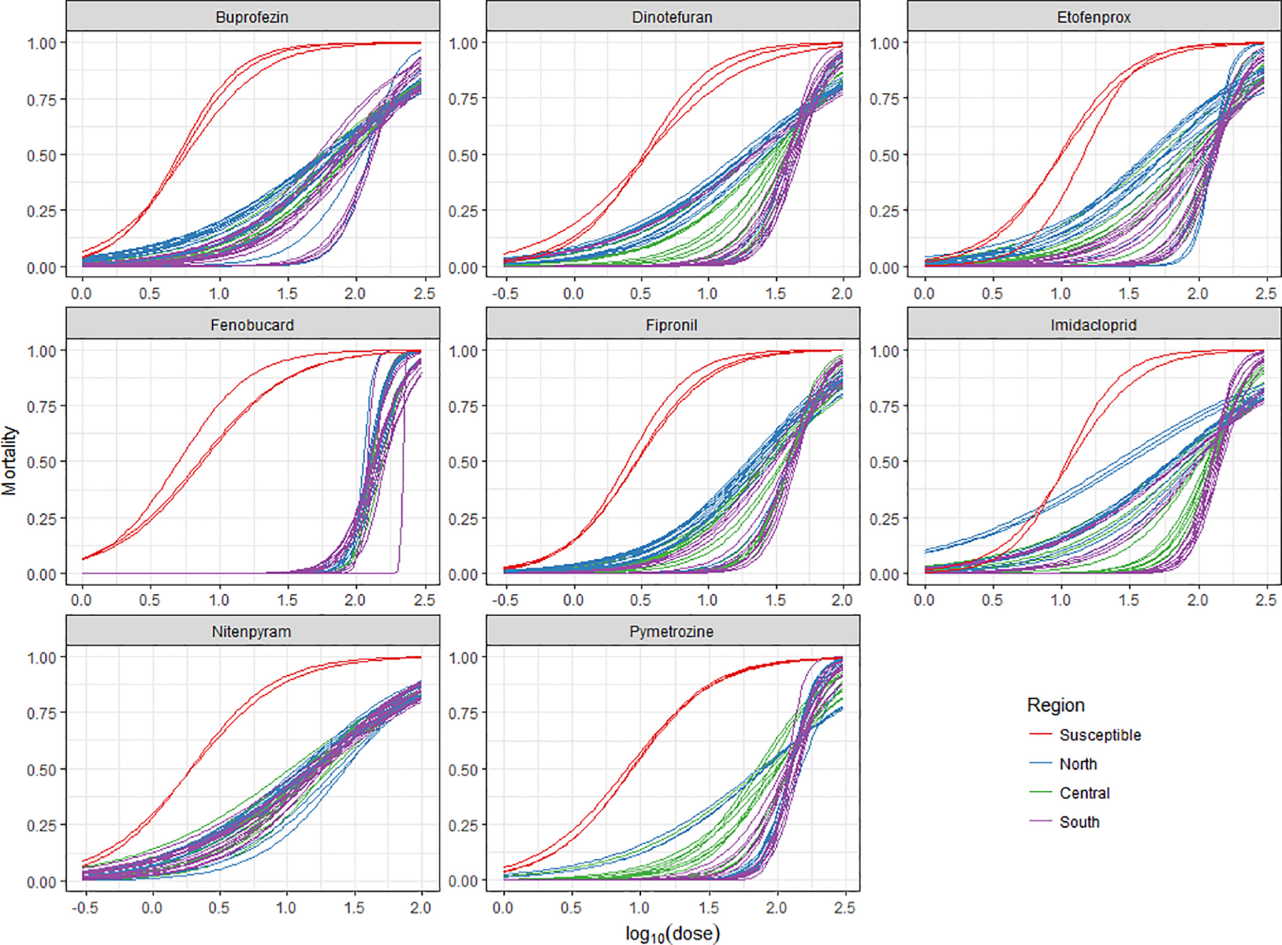
Dose-response curves for the eight insecticides investigated in Vietnam 2015–17.

We used an IRAC susceptibility test method for investigated insecticide resistance of two BPH populations per year in the north, central and south of Vietnam, from 2015–2017. Fig 1 presents the results graphically. The LC_50_ values and slopes referred to below are calculated from these dose-response curves. The specific data for each insecticide are available in S1–S8 Tables. The susceptible laboratory population always had a lower LC_50_ than any of the field samples. Initially, the variation of data between years from each insecticide was compared. When there was no significant difference, the data was pooled for further analysis. Secondly, differences between the regions were analyzed and finally differences between seasons were compared.

### Resistance status in Vietnamese BPH populations

There was a 2.5-, 5.9-, 2.6-fold variation in the susceptibility of the three neonicotinoids dinotefuran, imidacloprid and nitenpyram, respectively, in BPHs populations from Vietnam. The most dinotefuran- and imidacloprid-resistant BPH populations were 14- and 13-fold resistant and were from the AnGiang province in the south (S3 and S6 Tables). The highest RI_50_ of 14 for nitenpyram was observed in the northern NamDinh province (S7 Table).

For dinotefuran there were significant differences of resistance among years and regions. Resistance was decreasing on a gradient: south > central > north. In 2015 dinotefuran had a higher LC_50_ in the south-central (LC_50_ AVG = 40.53±1.43 mg mL^-1^) compared to in the north (AVG = 18.93±1.65 mg mL^-1^, P<0.001), and the slope of the response was steeper (north slope AVG = 0.86±0.06, south-central slope AVG = 3.05±0.37, P<0.001). Both LC_50_ (2016 AVG = 39.31±1.22 mg mL^-1^, 2017 AVG = 24.63±1.59 mg mL^-1^, P<0.001) and slope (2016 AVG = 2.36±0.24, 2017 AVG = 0.95±0.06, P<0.001) decreased from 2016 to 2017.

There were significant differences of resistance for imidacloprid among years and regions. While susceptibility of imidacloprid remained the same in the north (LC_50_ AVG = 60.77±3.32 mg mL^-1^, slope AVG = 0.85±0.04), LC_50_ dropped from 2015–16 (LC_50_ AVG = 129.05±3.69 mg mL^-1^, slope AVG = 3.65±0.41) to 2017 in the south-central region (LC_50_ AVG = 81.31±5.14 mg mL^-1^, P<0.001) and the slope decreased (slope AVG = 0.99±0.06, P<0.001). LC_50_ in the south-central in 2017 did not reach the same level as in the north (P<0.001); the slopes, however, became the same (P = 0.07).

For nitenpyram there were no differences of resistance between field samples; average LC_50_ was 15.97±0.45 mg mL^-1^, and the average slope was 0.91±0.02.

There was a 2.4-fold variation in the susceptibility of buprofenzin in BPHs populations from Vietnam. The most buprofenzin-resistant BPH populations (RI_50_ = 24) were from the southern AnGiang and SocTrang provinces (S1 Table). For buprofezin, there were significant differences of resistance among regions only. Buprofezin had a higher LC_50_ (AVG = 280.63±12.04 mg mL^-1^) in the south compared to that of the north-central (AVG = 234.40±8.03 mg mL^-1^) (P<0.001), and the slope of the response was steeper in the Mekong River populations (south AVG slope = 1.33±0.08, north-central AVG slope = 1.04±0.04, P = 0.001).

There was a 3.3-fold variation in the susceptibility of etofenprox in BPHs populations from Vietnam. The most etofenprox-resistant BPH populations (RI_50_ = 12) was found in populations collected in the southern (S2 Table). For etofenprox there were significant differences among years and regions. LC_50_ increased from the first to the second growing season, mostly with a concomitant increase in slope (not shown).

There was a 1.5-fold variation in the susceptibility of fenobucarb in BPHs populations from Vietnam. The most fenobucarb-resistant BPH population (RI_50_ = 25) was found in the southern AnGiang province (S4 Table). For fenobucard there were no significant differences in susceptibility, AVG LC_50_ was 690.31±12.84 mg L^-1^ and the average slope was 4.52±0.25.

There was a 2.3-fold variation in the susceptibility of fipronil in BPHs populations from Vietnam. The most fipronil-resistant population (RI_50_ = 16) was from the southern AnGiang province (S5 Table). For fipronil there were significant differences among years and regions. In the north, 2016 stood out with a higher LC_50_ in 2016 (AVG = 36.19±2.64 mg mL^-1^, slope = 1.76±0.34) than that in the year before (LC_50_ AVG = 21.22±1.46 mg mL^-1^, slope AVG = 1.19±0.09, P<0.001) and year after (LC_50_ AVG = 21.98±1.51 mg mL^-1^, slope AVG = 1.19±0.09, P<0.001), while the slope remained the same. In the south-central LC_50_ dropped from 2015–16 (LC_50_ AVG 40.82±0.98 mg mL^-1^, slope AVG = 3.09±0.25) to 2017 (AVG LC_50_ = 28.16±1.29 mg mL^-1^, P<0.001) and the slope decreased (AVG slope = 1.39±0.09, P<0.001).

There was a 2.1-fold variation in the susceptibility of pymetrozine in BPH populations from Vietnam. The most pymetrozine-resistant BPH population (RI_50_ = 17) was found in the northern VinhPhuc province (S8 Table). For pymetrozine there were significant differences only among regions, with a lower LC_50_ in the central region (LC_50_ AVG = 322.25±13.16 mg mL^-1^) compared to the north (LC_50_ AVG = 396.47±11.03 mg mL^-1^, P<0.001) and the south (LC_50_ AVG = 396.59±11.48, P<0.001). Likewise, the slope from the central region (AVG slope = 1.69±0.14) was less step in comparison to north (AVG slope = 2.87±0.33, P<0.001) and south (AVG slope = 3.03±0.33, P<0.001).

### Cross-resistance of insecticides

The cross-resistance pattern of the eight insecticides in this survey were investigated by pairwise comparison and calculation of correlation coefficient (Table 1).

Noteworthy is the correlation of fipronil toxicity with dinotefuran (0.92; P<0.00), etofenprox (0.73, P<0.00), buprofezin (0.51, P<0.00), imidacloprid (0.47, P<0.01) (Table 1). Likewise, imidacloprid show correlation to dinotefuran (0.47, P<0.01) and etofenprox (0.41, P<0.05). The correlation of imidacloprid and dinotefuran is the only correlation among neonicotinoids in this investigation, where nitenpyram is showing no correlation to any other insecticides. Pymetrozine and fenobucarb was also without correlation to any of the investigated insecticides (Table 1).

**Table 1:** Correlation matrix evaluating cross-resistance pattern of BPH populations from Vietnam.

Insecticide	Buprofezin	Dinotefuran	Etofenprox	Fenobucarb	Fipronil	Imidacloprid	Nitenpyram	Pymetrozin
Buprofezin		0.47	0.62	0.32	0.51	0.31	0.04	0.26
Dinotefuran	0.01		0.79	0.21	0.92	0.47	0.091	0.15
Etofenprox	0.00	0.00		0.27	0.73	0.41	0.062	0.24
Fenobucarb	0.39	1.00	0.77		0.19	0.15	0.17	0.18
Fipronil	0.00	0.00	0.00	1.00		0.47	0.20	-0.02
Imidacloprid	0.45	0.01	0.05	1.00	0.01		0.28	-0.19
Nitenpyram	1.00	1.00	1.00	1.00	1.00	0.62		-0.29
Pymetrozin	0.86	1.00	1.00	1.00	1.00	1.00	0.60	

Numbers in the matrix above the diagonal are correlation coefficients. Numbers below the diagonal are p-values.

### Combining LC_50_ and the slope of the toxicology regression line

**Fig 2:**
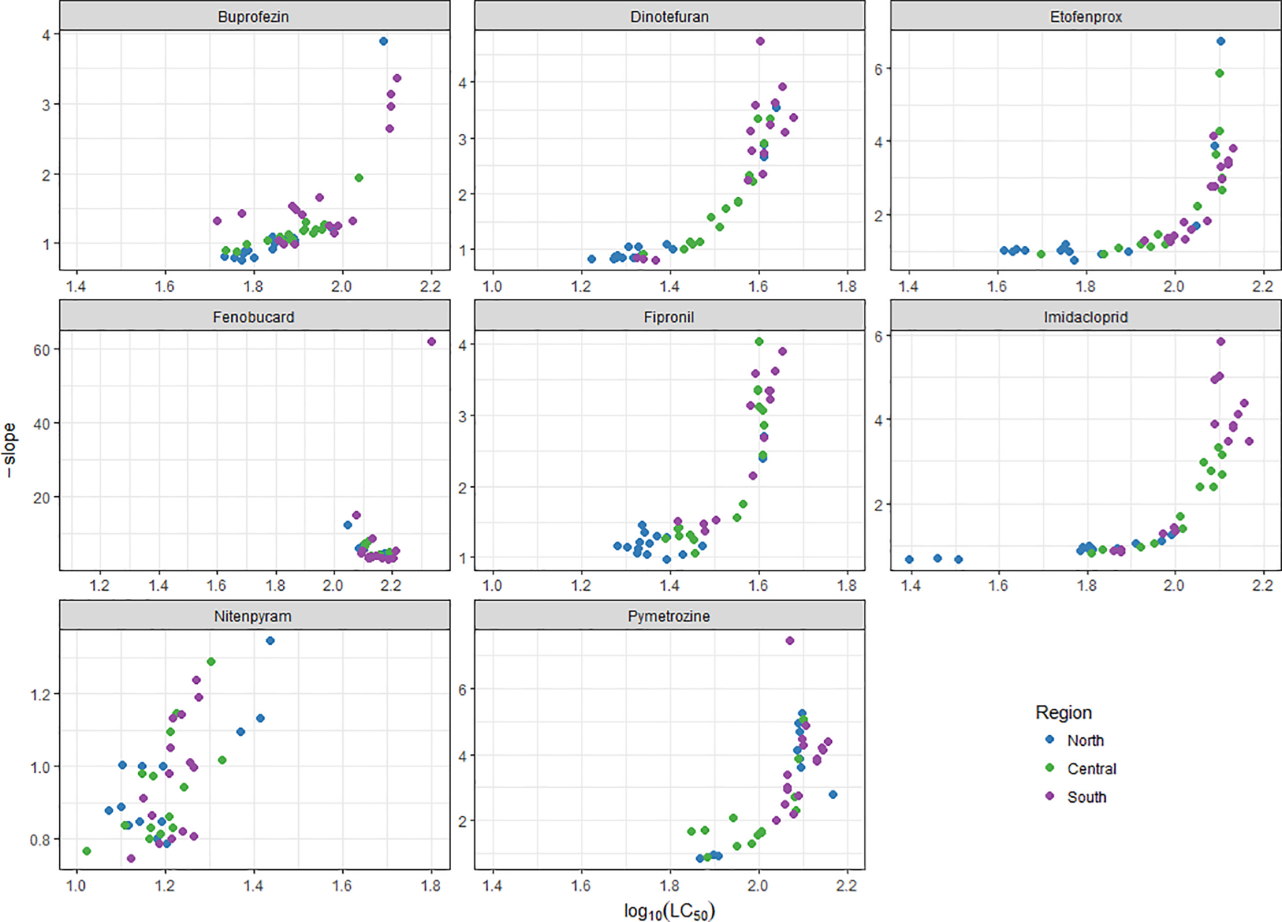
Correlation of LC_50_ and slope of BPH populations from three regions of Vietnam.

Comparisons of corresponding LC_50_ values and the slope of the dose-response regression lines are presented in Fig 2. An increasing LC_50_ value is linked to an increasing slope for buprofezin, dinotefuran, etofenprox, fipronil, imidacloprid and pymetrozine. The LC_50_’s and slopes appears as biphasic. There is an initial phase with low LC_50_ and low slope, which could be a visualization of the phase where selection initially have decreased the slope substantially and increased the LC_50_ slightly, which is an indication of a heterogenous population with a mixture of resistant phenotypes. In the second phase, resistance selection is reaching the stage where LC_50_ has increased and the slope is increasing as well, which is observed for buprofezin, dinotefuran, etofenprox, fipronil, imidacloprid and pymetrozine (Fig 2). This is indicative of the populations getting more homogenous. For nitenpyram the data seems to be random distributed and there is no correlation (Fig 2). The fenobucarb data show very little variation in slope and there is no correlation (Fig 2).

## Discussion

The control of the BPH has relied on various insecticides throughout Asia. Initially, highly persistent organochlorines such as DDT (dichlordiphenyltrichlorethan) and BHC (benzene hexachloride) were used, but these insecticides have been banned since the 1970s due to environmental impact. Subsequently, the organophosphates and carbamates were widely used as control agents against BPH, but were replaced due to resistance. During the last decades pyrethroid, neonicotinoid, insect growth regulator and phenylpyrazole insecticides have been widely applied to control BPH. The extensive use of these insecticides evolved resistant populations of BPH and populations with high levels of resistance to the major classes of insecticides has emerged throughout Asia [19, 21, 27, 28].

The Vietnam BPH populations have moderate anticholinesterase resistance levels, which are similar to the stable medium resistance levels observed in other Asian countries [12, 21, 23, 29–32]. An earlier study from Vietnam showed a much higher level of variation among populations for fenobucarb toxicity [22], which could be attributed to the use of topical application bioassay, which focuses more on intrinsic toxicological properties of the insecticide rather than the interaction of the pest with the insecticide in a simulated exposure situation. Anticholinesterase insecticides should be used with caution since resistance is prominent in many Asian countries. Additionally, it is important to be aware of the correlations between anticholinesterases and neonicotinoids, indicating cross-resistance possibly caused by detoxifying enzymes.

Pyrethroid resistance in the present study was low like in studies from China, Korea and Japan [29–32]. The pyrethroid etofenprox is of interest in the context of IPM programs in rice since it is relatively safe to some BPHs predators [33–36]. Sublethal doses of etofenprox did not increase fecundity of BPH [37] and it is relatively safe to its natural enemies. The resurgence risk is low and it could be a good choice for IPM programs.

Neonicotinoid resistance emerged across Asia in 2003–2006. Imidacloprid resistance, even at very high levels, was implied in samples from many Asian countries and there was a positive cross-resistance to thiamethoxam, but no cross-resistance to dinotefuran [12, 21, 30, 31, 38–40]. The latest Chinese survey covering 2012–16 including 70 populations showed extremely high resistance levels up to 8,500-fold, whereas populations were susceptible or moderately resistant to the nitromethylene neonicotinoid nitenpyram [23, 41]. The present investigation of the situation in Vietnam show, in contrast to an ealier survey [22], that populations are susceptible or lowly resistant to neonicotinoids and they could be used to control BPH. The discrepancy to the abovementioned investigation form Vietnam is most likely due the very different methods used; topical application and rice seedling dipping, respectively. The regional history of resistance development to this mode of action indicates a high potential for resistance development. The potential for resistance development in Vietnam is also indicated by the relative low slopes found in many Vietnamese populations for dinotefuran, imidacloprid and nitenpyram.

Resistance to phenyl pyrazole insecticides, e.g. fipronil and ethiprole, were low like in other studies [12, 22, 29, 31]. A high level of resistance was found in populations from Thailand and China [19, 36, 40] and there was no cross-resistance to imidacloprid, dinotefuran and etofenprox [19]. A Vietnamese population from 2008 was 100-fold resistant to fipronil [40], but our investigation indicates that fipronil will show efficacy against BPH.

The chitin synthesis inhibitor buprofezin is a thiadiazine insecticide that is especially effective against Homoptera (Hemiptera) pests like BPH [42–44]. A low to moderate level of resistance were found in our Vietnamese survey, like it has been seen in Chinese surveys [15]. In China buprofezin continue its increasing resistance trend [21, 23, 30], whereas in Vietnamese populations 2015–2017 there is no trend towards a high level of resistance.

The current situation in Vietnam is that BPH populations are low to moderately resistant to pymetrozine and there are no indications of problems with its use. Pymetrozine, an azomethine pyridine insecticide, is extremely effective against sucking pest insects [45]. The moderate level of pymetrozine resistance without cross-resistance observed in China [21, 46] was decreasing in 2014–15 [23].

The hypothesis of a gradient of susceptibility from the Red River Delta in the north to the Mekong River Delta in the south of Vietnam is not rejected. Mostly the highest resistance index is found in the south and the general trend is following the hypothesis. There was a significant geographic difference in toxicity for dinotefuran, imidacloprid, buprofezin, etofenprox and fipronil. Nitenpyram and fenobucarb did not show any geographic significant differences, whereas pymetrozine was unique with the lowest LC_50_ values in the central provinces.

The hypothesized decreased susceptibility of BPH populations from 2015 to 2017 and from spring to autumn was largely not shown. In 2015, dinotefuran had a higher LC_50_ in the south and central provinces compared to that in the north. Dinotefuran LC_50_ decreased in 2016 and 2017. Etofenprox toxicity increased significantly in some years in some regions and also showed some seasonal differences, but generally there was not a trend of decreasing susceptibility. For fipronil, in 2016 in the northern provinces, LC_50_ was significantly increased compared to 2015, but it decreased again in 2017. A similar decreasing fipronil toxicity was observed in the central and southern provinces in 2017.

Insecticide resistance in Vietnam is not at an alarming level and we did not even identify a single population with a very high level of resistance to any of the insecticides. This could be the result of a successful campaign to improve farmers’ pest management decisions in Vietnam, where print materials, such as pamphlets and posters, billboards, and radio and TV programs were designed to motivate farmers to avoid using insecticides in the first 40 days after sowing. These programs had positive effects on farmers’ beliefs, attitudes, and practices, reducing insecticide sprays by more than 50% [47].

The eight insecticides investigated in BPH populations in Vietnam are at different stages of resistance development. Anticholinesterase resistance was probably established many decades ago and is at a homogenous stable level reflected by the low level of variation found on fenobucarb. Nitenpyram is at the other end of resistance development, mostly showing the natural variation of susceptibility. For fipronil, etofenprox, buprofezin, dinotefuran and imidacloprid we found heterogenous populations in the early stages of resistance selection increased LC_50_ and low slope, as well as more homogenous populations, which have advanced in their resistance selection with further increased LC_50_ values and steeper slopes.

The cross-resistance pattern of this survey showed that nitenpyram, pymetrozine and fenobucarb was not involved in any relationships. The cross-resistance of fipronil, etofenprox, buprofezin and the neonicotinoids dinotefuran and imidaclopird in this investigation should be noted and the background of this tolerance to multiple insecticides of different modes of action should be investigated.

In conclusion, the cross-resistance pattern of BPH populations in Vietnam, where insecticides with different modes of action show correlation, indicate that insecticides should be used with caution. There could be a buildup of a troublesome general metabolic resistance, which alone or in combination with the emergence of target-site resistance mutation will cause control problems. Vietnam is not a focal point of insecticide resistance origin spreading throughout South-East Asia. The results will be beneficial for development of resistance management strategies to prevent and delay development of insecticide resistance in BPH not only from Vietnam, but also from countries in the more northern Asian regions due the migration of BPH from tropical Vietnam.

## Supporting information

S1 Table Results of the bioassay with buprofenzin of BPH populations from North, Central and South Vietnam. RI_50_ were calculated by dividing LC_50_ with AVG LC_50_ (16.90) of the susceptible population. Year-1 and year-2 signify summer-autumn and winter-spring sampling of BPH.
(DOCX)

S2 Table Results of the bioassay with etofenprox of BPH populations from North, Central and South Vietnam. RI_50_ were calculated by dividing LC_50_ with AVG LC_50_ (22.80) of the susceptible population. Year-1 and year-2 signify summer-autumn and winter-spring sampling of BPH.
(DOCX)

S3 Table Results of the bioassay with dinotefuran of BPH populations from North, Central and South Vietnam. RI_50_ were calculated by dividing LC_50_ with AVG LC_50_ (3.42) of the susceptible population. Year-1 and year-2 signify summer-autumn and winter-spring sampling of BPH.
(DOCX)

S4 Table Results of the bioassay with fenobucarb of BPH populations from North, Central and South Vietnam. RI_50_ were calculated by dividing LC_50_ with AVG LC_50_ (32.81) of the susceptible population. Year-1 and year-2 signify summer-autumn and winter-spring sampling of BPH.
(DOCX)

S5 Table Results of the bioassay with fipronil of BPH populations from North, Central and South Vietnam. RI_50_ were calculated by dividing LC_50_ with AVG LC_50_ (2.74) of the susceptible population. Year-1 and year-2 signify summer-autumn and winter-spring sampling of BPH.
(DOCX)

S6 Table Results of the bioassay with imidacloprid of BPH populations from North, Central and South Vietnam. RI_50_ were calculated by dividing LC_50_ with AVG LC_50_ (11.33) of the susceptible population. Year-1 and year-2 signify summer-autumn and winter-spring sampling of BPH.
(DOCX)

S7 Table Results of the bioassay with nitenpyram of BPH populations from North, Central and South Vietnam. RI_50_ were calculated by dividing LC_50_ with AVG LC_50_ (1.90) of the susceptible population. Year-1 and year-2 signify summer-autumn and winter-spring sampling of BPH.
(DOCX)

S8 Table Results of the bioassay with pymetrozine of BPH populations from North, Central and South Vietnam. RI_50_ were calculated by dividing LC_50_ with AVG LC_50_ (26.34) of the susceptible population. Year-1 and year-2 signify summer-autumn and winter-spring sampling of BPH.
(DOCX)
