# Supplementary material for: Variation in susceptibility of eight insecticides in the brown planthopper Nilaparvata lugens in three regions of Vietnam 2015-2017
Source: PLoS One. 2018 Oct 5;13(10):e0204962. doi: 10.1371/journal.pone.0204962 (PMC6173402; doi:10.1371/journal.pone.0204962)
Supplement: S1 Table — RI50 were calculated by dividing LC50 with AVG LC50 (16.90) of the susceptible population. Year-1 and year-2 signify summer-autumn and winter-spring sampling of BPH. (DOCX) [file pone.0204962.s001.docx]

**S1 Table. Results of the bioassay with buprofenzin of BPH populations from North, Central and South Vietnam.** RI_50_ were calculated by dividing LC_50_ with AVG LC_50_ (16.90) of the susceptible population. Year-1 and year-2 signify summer-autumn and winter-spring sampling of BPH.

| Locality | Year-Season | LC_50_ ± SE | Slope ± SE | RI_50_ |
| --- | --- | --- | --- | --- |
|  |  | mg L^-1^ |  |  |
| Susceptible | 2015 | 16.82 ± 2.35 | 1.98 ± 0.47 |  |
|  | 2016 | 15.92 ± 2.15 | 2.13 ± 0.52 |  |
|  | 2017 | 17.96 ± 2.78 | 1.60 ± 0.34 |  |
| North |  |  |  |  |
| HaiPhong | 2015-1 | 190.26 ± 43.67 | 0.78 ± 0.14 | 11 |
|  | 2015-2 | 191.01 ± 41.54 | 0.86 ± 0.15 | 11 |
|  | 2016-1 | 222.17 ± 46.28 | 0.94 ± 0.21 | 13 |
|  | 2016-2 | 247.43 ± 47.87 | 1.11 ± 0.27 | 15 |
|  | 2017-1 | 196.69 ± 41.4 | 0.92 ± 0.17 | 12 |
|  | 2017-2 | 325.91 ± 55.43 | 1.77 ± 0.67 | 19 |
| NamDinh | 2015-1 | 201.59 ± 45.58 | 0.80 ± 0.15 | 12 |
|  | 2015-2 | 191.64 ± 40.68 | 0.89 ± 0.17 | 11 |
|  | 2016-1 | 240.8 ± 46.58 | 1.12 ± 0.29 | 14 |
|  | 2016-2 | 299.86 ± 56.39 | 1.29 ± 0.39 | 18 |
|  | 2017-1 | 250.17 ± 49.76 | 1.07 ± 0.23 | 15 |
|  | 2017-2 | 383.04 ± 43.42 | 2.79 ± 1.40 | 23 |
| VinhPhuc | 2015-1 | 183.38 ± 41.26 | 0.81 ± 0.14 | 11 |
|  | 2015-2 | 185.06 ± 39.53 | 0.89 ± 0.16 | 11 |
|  | 2016-1 | 221.49 ± 42.23 | 1.12 ± 0.26 | 13 |
|  | 2016-2 | 225.42 ± 44.99 | 1.03 ± 0.24 | 13 |
|  | 2017-1 | 173.35 ± 38.64 | 0.82 ± 0.14 | 10 |
|  | 2017-2 | 263.85 ± 47.52 | 1.36 ± 0.36 | 16 |
| Central |  |  |  |  |
| Hue | 2015-1 | 262.65 ± 49.26 | 1.25 ± 0.31 | 16 |
|  | 2015-2 | 261.66 ± 49.39 | 1.23 ± 0.31 | 15 |
|  | 2016-1 | 246.27 ± 48.07 | 1.09 ± 0.27 | 15 |
|  | 2016-2 | 325.9 ± 55.43 | 1.77 ± 0.67 | 19 |
|  | 2017-1 | 245.16 ± 48.07 | 1.08 ± 0.22 | 15 |
|  | 2017-2 | 185.05 ± 39.53 | 0.89 ± 0.16 | 11 |
| NgheAn | 2015-1 | 241.61 ± 46.01 | 1.18 ± 0.28 | 14 |
|  | 2015-2 | 231.01 ± 44.51 | 1.12 ± 0.25 | 14 |
|  | 2016-1 | 234.73 ± 46.26 | 1.05 ± 0.23 | 14 |
|  | 2016-2 | 263.85 ± 47.52 | 1.36 ± 0.36 | 16 |
|  | 2017-1 | 194.54 ± 38.98 | 1.00 ± 0.18 | 12 |
|  | 2017-2 | 175.2 ± 37.06 | 0.90 ± 0.16 | 10 |
| PhuYen | 2015-1 | 278.8 ± 53.16 | 1.26 ± 0.34 | 17 |
|  | 2015-2 | 291.81 ± 56.36 | 1.35 ± 0.43 | 17 |
|  | 2016-1 | 275 ± 52.56 | 1.19 ± 0.32 | 16 |
|  | 2017-1 | 286.77 ± 54.09 | 1.26 ± 0.31 | 17 |
|  | 2017-2 | 216.55 ± 42.23 | 1.07 ± 0.21 | 13 |
| South |  |  |  |  |
| AnGiang | 2015-1 | 247.67 ± 40.15 | 1.53 ± 0.35 | 15 |
|  | 2015-2 | 275.58 ± 42.82 | 1.70 ± 0.43 | 16 |
|  | 2016-1 | 387.64 ± 50.96 | 2.27 ± 0.96 | 23 |
|  | 2016-2 | 408.36 ± 49.29 | 2.68 ± 1.15 | 24 |
|  | 2017-1 | 334.36 ± 62.56 | 1.39 ± 0.43 | 20 |
|  | 2017-2 | 249.29 ± 49.95 | 1.02 ± 0.20 | 15 |
| LongAn | 2015-1 | 166.5 ± 28.2 | 1.36 ± 0.29 | 10 |
|  | 2015-2 | 242.46 ± 38.37 | 1.60 ± 0.40 | 14 |
|  | 2016-1 | 308.77 ± 62.47 | 1.22 ± 0.42 | 18 |
|  | 2016-2 | 356.93 ± 69.3 | 1.61 ± 0.73 | 21 |
|  | 2017-1 | 298.77 ± 56.95 | 1.32 ± 0.37 | 18 |
|  | 2017-2 | 229.14 ± 45.02 | 1.07 ± 0.21 | 14 |
| SocTrang | 2015-1 | 188.51 ± 30.63 | 1.47 ± 0.32 | 11 |
|  | 2015-2 | 256.63 ± 43.44 | 1.45 ± 0.35 | 15 |
|  | 2016-1 | 408.67 ± 49.39 | 2.68 ± 1.15 | 24 |
|  | 2016-2 | 391.82 ± 47.48 | 2.53 ± 1.02 | 23 |
|  | 2017-1 | 311.74 ± 57.36 | 1.31 ± 0.35 | 18 |
|  | 2017-2 | 235.37 ± 46.92 | 1.02 ± 0.20 | 14 |
