# Supplementary material for: Variation in susceptibility of eight insecticides in the brown planthopper Nilaparvata lugens in three regions of Vietnam 2015-2017
Source: PLoS One. 2018 Oct 5;13(10):e0204962. doi: 10.1371/journal.pone.0204962 (PMC6173402; doi:10.1371/journal.pone.0204962)
Supplement: S2 Table — RI50 were calculated by dividing LC50 with AVG LC50 (22.80) of the susceptible population. Year-1 and year-2 signify summer-autumn and winter-spring sampling of BPH. (DOCX) [file pone.0204962.s002.docx]

**S2 Table. Results of the bioassay with etofenprox of BPH populations from North, Central and South Vietnam.** RI_50_ were calculated by dividing LC_50_ with AVG LC_50_ (22.80) of the susceptible population. Year-1 and year-2 signify summer-autumn and winter-spring sampling of BPH.

| Locality | Year-Season | LC_50_ ± SE | Slope ± SE | RI_50_ |
| --- | --- | --- | --- | --- |
|  |  | mg L^-1^ |  |  |
| Susceptible | 2015 | 19.39 ± 2.81 | 1.75 ± 0.41 |  |
|  | 2016 | 20.12 ± 3.04 | 1.55 ± 0.32 |  |
|  | 2017 | 28.88 ± 3.57 | 2.24 ± 0.66 |  |
| North |  |  |  |  |
| HaiPhong | 2015-1 | 87.58 ± 16.14 | 1.06 ± 0.19 | 4 |
|  | 2015-2 | 112.00 ± 20.38 | 1.10 ± 0.22 | 5 |
|  | 2016-1 | 136.83 ± 27.39 | 0.93 ± 0.18 | 6 |
|  | 2016-2 | 246.30 ± 32.28 | 3.89 ± 2.64 | 11 |
|  | 2017-1 | 86.43 ± 16.36 | 1.00 ± 0.17 | 4 |
|  | 2017-2 | 249.72 ± NA | 7.25 ± NA | 11 |
| NamDinh | 2015-1 | 91.71 ± 17.19 | 1.03 ± 0.18 | 4 |
|  | 2015-2 | 113.49 ± 19.88 | 1.19 ± 0.25 | 5 |
|  | 2016-1 | 156.51 ± 30.33 | 1.01 ± 0.21 | 7 |
|  | 2016-2 | 223.23 ± 42.31 | 1.70 ± 0.73 | 10 |
|  | 2017-1 | 115.8 ± 22.26 | 0.98 ± 0.19 | 5 |
|  | 2017-2 | 248.34 ± 29.23 | 3.64 ± 2.28 | 11 |
| VinhPhuc | 2015-1 | 86.43 ± 16.36 | 1.00 ± 0.17 | 4 |
|  | 2015-2 | 111.01 ± 20.9 | 1.02 ± 0.19 | 5 |
|  | 2016-1 | 118.67 ± 25.89 | 0.78 ± 0.14 | 5 |
|  | 2016-2 | 241.01 ± 25.98 | 2.78 ± 2.00 | 11 |
|  | 2017-1 | 82.58 ± 15.36 | 1.03 ± 0.18 | 4 |
|  | 2017-2 | 253.89 ± 98.99 | 5.79 ± 9.52 | 11 |
| Central |  |  |  |  |
| Hue | 2015-1 | 183.63 ± 32.47 | 1.46 ± 0.39 | 8 |
|  | 2015-2 | 251.76 ± 41.26 | 4.28 ± 3.25 | 11 |
|  | 2016-1 | 190.87 ± 35.72 | 1.19 ± 0.31 | 8 |
|  | 2016-2 | 264.41 ± 30.32 | 3.37 ± 1.41 | 12 |
|  | 2017-1 | 148.97 ± 27.56 | 1.11 ± 0.20 | 7 |
|  | 2017-2 | 256.02 ± 26.97 | 3.01 ± 1.29 | 11 |
| NgheAn | 2015-1 | 137.96 ± 27.95 | 0.93 ± 0.18 | 6 |
|  | 2015-2 | 248.01 ± 29.29 | 3.66 ± 2.31 | 11 |
|  | 2016-1 | 176.60 ± 33.12 | 1.13 ± 0.27 | 8 |
|  | 2016-2 | 248.34 ± 29.23 | 3.64 ± 2.28 | 11 |
|  | 2017-1 | 100.01 ± 19.80 | 0.93 ± 0.15 | 4 |
|  | 2017-2 | 244.04 ± NA | 6.96 ± NA | 11 |
| PhuYen | 2015-1 | 225.18 ± 41.33 | 2.25 ± 1.63 | 10 |
|  | 2015-2 | 264.51 ± 30.28 | 3.37 ± 1.4 | 12 |
|  | 2016-1 | 255.31 ± 27.89 | 2.67 ± 1.41 | 11 |
|  | 2017-1 | 168.45 ± 30.92 | 1.19 ± 0.25 | 7 |
|  | 2017-2 | 246.20 ± 25.66 | 2.80 ± 1.19 | 11 |
| South |  |  |  |  |
| AnGiang | 2015-1 | 217.85 ± 41.28 | 1.59 ± 0.61 | 10 |
|  | 2015-2 | 263.59 ± 31.43 | 3.49 ± 1.47 | 12 |
|  | 2016-1 | 270.82 ± 36.36 | 3.81 ± 1.65 | 12 |
|  | 2016-2 | 254.66 ± 28.54 | 3.31 ± 1.41 | 11 |
|  | 2017-1 | 210.52 ± 40.23 | 1.33 ± 0.41 | 9 |
|  | 2017-2 | 246.23 ± 25.68 | 2.79 ± 1.18 | 11 |
| LongAn | 2015-1 | 171.37 ± 30.50 | 1.31 ± 0.3 | 8 |
|  | 2015-2 | 240.86 ± 25.93 | 2.79 ± 2.03 | 11 |
|  | 2016-1 | 271.14 ± 35.97 | 3.79 ± 1.61 | 12 |
|  | 2016-2 | 256.08 ± 26.93 | 2.99 ± 1.31 | 11 |
|  | 2017-1 | 192.93 ± 36.37 | 1.35 ± 0.38 | 8 |
|  | 2017-2 | 208.78 ± 31.70 | 1.81 ± 0.54 | 9 |
| SocTrang | 2015-1 | 199.23 ± 37.83 | 1.44 ± 0.44 | 9 |
|  | 2015-2 | 244.02 ± 36.57 | 4.14 ± 3.25 | 11 |
|  | 2016-1 | 264.21 ± 30.32 | 3.38 ± 1.42 | 12 |
|  | 2016-2 | 263.56 ± 31.39 | 3.49 ± 1.46 | 12 |
|  | 2017-1 | 194.94 ± 35.64 | 1.26 ± 0.32 | 9 |
|  | 2017-2 | 237.23 ± 60.30 | 1.82 ± 1.33 | 10 |
