# Supplementary material for: Variation in susceptibility of eight insecticides in the brown planthopper Nilaparvata lugens in three regions of Vietnam 2015-2017
Source: PLoS One. 2018 Oct 5;13(10):e0204962. doi: 10.1371/journal.pone.0204962 (PMC6173402; doi:10.1371/journal.pone.0204962)
Supplement: S3 Table — RI50 were calculated by dividing LC50 with AVG LC50 (3.42) of the susceptible population. Year-1 and year-2 signify summer-autumn and winter-spring sampling of BPH. (DOCX) [file pone.0204962.s003.docx]

**S3 Table. Results of the bioassay with dinotefuran of BPH populations from North, Central and South Vietnam.** RI_50_ were calculated by dividing LC_50_ with AVG LC_50_ (3.42) of the susceptible population. Year-1 and year-2 signify summer-autumn and winter-spring sampling of BPH.

| Locality | Season-year | LC_50_ ± SE | Slope ± SE | RI_50_ |
| --- | --- | --- | --- | --- |
|  |  | mg L^-1^ |  |  |
| Susceptible | 2015 | 3.54 ± 0.36 | 1.49 ± 0.20 |  |
|  | 2016 | 3.30 ± 0.28 | 1.71 ± 0.24 |  |
|  | 2017 | 3.44 ± 0.59 | 1.21 ± 0.22 |  |
| North |  |  |  |  |
| HaiPhong | 2015-1 | 19.65 ± 4.98 | 0.86 ± 0.18 | 6 |
|  | 2015-2 | 18.77 ± 4.95 | 0.84 ± 0.18 | 5 |
|  | 2016-1 | 21.34 ± 3.66 | 1.04 ± 0.20 | 6 |
|  | 2016-2 | 40.86 ± 4.83 | 2.89 ± 1.11 | 12 |
|  | 2017-1 | 18.77 ± 5.45 | 0.84 ± 0.20 | 5 |
|  | 2017-2 | 25.42 ± 7.22 | 1.00 ± 0.31 | 7 |
| NamDinh | 2015-1 | 20.82 ± 5.65 | 0.85 ± 0.19 | 6 |
|  | 2015-2 | 18.87 ± 4.96 | 0.88 ± 0.20 | 6 |
|  | 2016-1 | 24.62 ± 5.49 | 1.09 ± 0.31 | 7 |
|  | 2016-2 | 43.60 ± 5.00 | 3.54 ± 1.10 | 13 |
|  | 2017-1 | 21.13 ± 6.15 | 0.86 ± 0.22 | 6 |
|  | 2017-2 | 27.04 ± 8.00 | 1.01 ± 0.34 | 8 |
| VinhPhuc | 2015-1 | 19.04 ± 4.92 | 0.89 ± 0.20 | 6 |
|  | 2015-2 | 16.71 ± 4.06 | 0.83 ± 0.15 | 5 |
|  | 2016-1 | 20.24 ± 3.36 | 1.05 ± 0.20 | 6 |
|  | 2016-2 | 40.87 ± 4.87 | 2.67 ± 1.12 | 12 |
|  | 2017-1 | 18.95 ± 5.42 | 0.86 ± 0.20 | 6 |
|  | 2017-2 | 21.59 ± 6.18 | 0.89 ± 0.23 | 6 |
| Central |  |  |  |  |
| Hue | 2015-1 | 33.63 ± 6.68 | 1.73 ± 0.76 | 10 |
|  | 2015-2 | 39.52 ± 5.36 | 3.34 ± 1.86 | 12 |
|  | 2016-1 | 32.49 ± 6.63 | 1.40 ± 0.52 | 10 |
|  | 2016-2 | 40.85 ± 5.49 | 2.90 ± 1.25 | 12 |
|  | 2017-1 | 21.13 ± 6.53 | 0.86 ± 0.23 | 6 |
|  | 2017-2 | 28.18 ± 7.90 | 1.09 ± 0.38 | 8 |
| NgheAn | 2015-1 | 31.07 ± 5.42 | 1.59 ± 0.49 | 9 |
|  | 2015-2 | 37.75 ± 6.61 | 2.34 ± 1.69 | 11 |
|  | 2016-1 | 27.90 ± 5.91 | 1.13 ± 0.33 | 8 |
|  | 2016-2 | 35.72 ± 5.81 | 1.85 ± 0.73 | 10 |
|  | 2017-1 | 21.77 ± 6.53 | 0.91 ± 0.24 | 6 |
|  | 2017-2 | 27.04 ± 8.00 | 1.01 ± 0.34 | 8 |
| PhuYen | 2015-1 | 35.73 ± 5.27 | 1.86 ± 0.66 | 10 |
|  | 2015-2 | 42.07 ± 4.16 | 3.34 ± 0.96 | 12 |
|  | 2016-1 | 38.66 ± 5.49 | 2.22 ± 1.03 | 11 |
|  | 2017-1 | 21.13 ± 6.53 | 0.86 ± 0.23 | 6 |
|  | 2017-2 | 29.22 ± 8.96 | 1.13 ± 0.48 | 9 |
| South |  |  |  |  |
| AnGiang | 2015-1 | 42.25 ± 5.30 | 3.23 ± 1.20 | 12 |
|  | 2015-2 | 47.50 ± 5.08 | 3.36 ± 0.76 | 14 |
|  | 2016-1 | 44.87 ± 4.85 | 3.91 ± 1.03 | 13 |
|  | 2016-2 | 47.51 ± 5.60 | 3.36 ± 0.83 | 14 |
|  | 2017-1 | 23.22 ± 7.55 | 0.82 ± 0.23 | 7 |
|  | 2017-2 | 40.49 ± 5.57 | 2.35 ± 1.27 | 12 |
| LongAn | 2015-1 | 38.18 ± 3.31 | 3.12± 1.17 | 11 |
|  | 2015-2 | 44.87 ± 5.30 | 3.91 ± 1.12 | 13 |
|  | 2016-1 | 43.43 ± 5.70 | 3.62 ± 1.26 | 13 |
|  | 2016-2 | 40.10 ± 8.58 | 4.73 ± 3.56 | 12 |
|  | 2017-1 | 21.13 ± 6.77 | 0.86 ± 0.24 | 6 |
|  | 2017-2 | 38.31 ± 4.38 | 2.78 ± 1.43 | 11 |
| SocTrang | 2015-1 | 39.15 ± 4.14 | 3.58 ± 1.52 | 11 |
|  | 2015-2 | 45.69 ± 5.10 | 3.11 ± 0.79 | 13 |
|  | 2016-1 | 40.87 ± 4.17 | 2.72 ± 0.93 | 12 |
|  | 2016-2 | 43.43 ± 4.48 | 3.63 ± 0.99 | 13 |
|  | 2017-1 | 21.88 ± 6.93 | 0.82 ± 0.22 | 6 |
|  | 2017-2 | 37.51 ± 6.34 | 2.24 ± 1.54 | 11 |
