# Supplementary material for: Variation in susceptibility of eight insecticides in the brown planthopper Nilaparvata lugens in three regions of Vietnam 2015-2017
Source: PLoS One. 2018 Oct 5;13(10):e0204962. doi: 10.1371/journal.pone.0204962 (PMC6173402; doi:10.1371/journal.pone.0204962)
Supplement: S4 Table — RI50 were calculated by dividing LC50 with AVG LC50 (32.81) of the susceptible population. Year-1 and year-2 signify summer-autumn and winter-spring sampling of BPH. (DOCX) [file pone.0204962.s004.docx]

**S4 Table. Results of the bioassay with fenobucarb of BPH populations from North, Central and South Vietnam.** RI_50_ were calculated by dividing LC_50_ with AVG LC_50_ (32.81) of the susceptible population. Year-1 and year-2 signify summer-autumn and winter-spring sampling of BPH.

| Locality | Year-Season | LC_50_ ± SE | Slope ± SE | RI_50_ |
| --- | --- | --- | --- | --- |
|  |  | mg L^-1^ |  |  |
| Susceptible | 2015 | 25.16 ± 2.91 | 1.67 ± 0.24 |  |
|  | 2016 | 37.69 ± 4.80 | 1.34 ± 0.18 |  |
|  | 2017 | 35.57 ± 4.63 | 1.31 ± 0.17 |  |
| North |  |  |  |  |
| HaiPhong | 2015-1 | 717.84 ± 90.87 | 4.41 ± 1.47 | 22 |
|  | 2015-2 | 568.32 ± 348.72 | 10.81 ± 51.8 | 17 |
|  | 2016-1 | 697.43 ± 79.15 | 4.09 ± 1.35 | 21 |
|  | 2016-2 | 674.97 ± 72.07 | 3.89 ± 1.32 | 21 |
|  | 2017-1 | 583.83 ± 328.38 | 8.92 ± 32.4 | 18 |
|  | 2017-2 | 611.13 ± NA | 6.91 ± NA | 19 |
| NamDinh | 2015-1 | 743.47 ± 101.16 | 4.69 ± 1.53 | 23 |
|  | 2015-2 | 693.67 ± 82.54 | 4.19 ± 1.44 | 21 |
|  | 2016-1 | 695.57 ± 81.34 | 4.15 ± 1.40 | 21 |
|  | 2016-2 | 695.48 ± 81.15 | 4.15 ± 1.40 | 21 |
|  | 2017-1 | 599.81 ± 273.36 | 8.80 ± 22.1 | 18 |
|  | 2017-2 | 612.57 ± NA | 7.96 ± NA | 19 |
| VinhPhuc | 2015-1 | 611.4 ± NA | 8.00 ± NA | 19 |
|  | 2015-2 | 548.47 ± NA | 12.86 ± NA | 17 |
|  | 2016-1 | 677.52 ± 69.78 | 3.8 ± 1.26 | 21 |
|  | 2016-2 | 656.05 ± 62.76 | 3.6 ± 1.20 | 20 |
|  | 2017-1 | 615.44 ± 606.5 | 6.66 ± 31.77 | 19 |
|  | 2017-2 | 606.55 ± NA | 7.16 ± NA | 18 |
| Central |  |  |  |  |
| Hue | 2015-1 | 604.09 ± NA | 6.28 ± NA | 18 |
|  | 2015-2 | 677.58 ± 69.58 | 3.8 ± 1.25 | 21 |
|  | 2016-1 | 717.95 ± 91.05 | 4.41 ± 1.47 | 22 |
|  | 2016-2 | 697.4 ± 79.23 | 4.09 ± 1.35 | 21 |
|  | 2017-1 | 585.72 ± NA | 11.84 ± NA | 18 |
|  | 2017-2 | 559.46 ± NA | 19.57 ± NA | 17 |
| NgheAn | 2015-1 | 625.91 ± 148.81 | 5.28 ± 5.66 | 19 |
|  | 2015-2 | 627.97 ± 129.27 | 5.18 ± 4.73 | 19 |
|  | 2016-1 | 658.58 ± 60.70 | 3.50 ± 1.14 | 20 |
|  | 2016-2 | 678.07 ± 69.25 | 3.79 ± 1.23 | 21 |
|  | 2017-1 | 650.48 ± 192.45 | 6.10 ± 6.84 | 20 |
|  | 2017-2 | 612.78 ± 499.48 | 9.18 ± 36.83 | 19 |
| PhuYen | 2015-1 | 677.46 ± 69.7 | 3.80 ± 1.25 | 21 |
|  | 2015-2 | 673.12 ± 73.46 | 3.94 ± 1.35 | 21 |
|  | 2016-1 | 695.46 ± 81.16 | 4.15 ± 1.40 | 21 |
|  | 2017-1 | 581.14 ± NA | 14.61 ± NA | 18 |
|  | 2017-2 | 772.76 ± 114.79 | 5.04 ± 1.62 | 24 |
| South |  |  |  |  |
| AnGiang | 2015-1 | 656.1 ± 62.65 | 3.59 ± 1.19 | 20 |
|  | 2015-2 | 675.23 ± 71.57 | 3.88 ± 1.30 | 21 |
|  | 2016-1 | 795.14 ± 76.99 | 3.39 ± 0.67 | 24 |
|  | 2016-2 | 733.39 ± 71.34 | 3.52 ± 0.83 | 22 |
|  | 2017-1 | 567.02 ± NA | 21.01 ± NA | 17 |
|  | 2017-2 | 809.56 ± 132.16 | 5.46 ± 1.75 | 25 |
| LongAn | 2015-1 | 618.48 ± 99.74 | 4.70 ± 3.65 | 19 |
|  | 2015-2 | 660.86 ± 58.77 | 3.39 ± 1.09 | 20 |
|  | 2016-1 | 697.86 ± 78.79 | 4.08 ± 1.33 | 21 |
|  | 2016-2 | 764.34 ± 70.18 | 3.07 ± 0.65 | 23 |
|  | 2017-1 | 590.53 ± 426.74 | 15.82 ± 68.7 | 18 |
|  | 2017-2 | 556.67 ± NA | 24.59 ± NA | 17 |
| SocTrang | 2015-1 | 629.59 ± 121.09 | 5.12 ± 4.31 | 19 |
|  | 2015-2 | 658.59 ± 60.68 | 3.50 ± 1.14 | 20 |
|  | 2016-1 | 695.51 ± 81.16 | 4.15 ± 1.40 | 21 |
|  | 2016-2 | 695.41 ± 81.21 | 4.15 ± 1.40 | 21 |
|  | 2017-1 | 780.81 ± NA | 25.09 ± NA | 24 |
|  | 2017-2 | 768.82 ± 117.65 | 5.08 ± 1.71 | 23 |
