# Supplementary material for: Variation in susceptibility of eight insecticides in the brown planthopper Nilaparvata lugens in three regions of Vietnam 2015-2017
Source: PLoS One. 2018 Oct 5;13(10):e0204962. doi: 10.1371/journal.pone.0204962 (PMC6173402; doi:10.1371/journal.pone.0204962)
Supplement: S5 Table — RI50 were calculated by dividing LC50 with AVG LC50 (2.74) of the susceptible population. Year-1 and year-2 signify summer-autumn and winter-spring sampling of BPH. (DOCX) [file pone.0204962.s005.docx]

**S5 Table. Results of the bioassay with fipronil of BPH populations from North, Central and South Vietnam.** RI_50_ were calculated by dividing LC_50_ with AVG LC_50_ (2.74) of the susceptible population. Year-1 and year-2 signify summer-autumn and winter-spring sampling of BPH.

| Locality | Year-Season | LC_50_ ± SE | Slope ± SE | RI_50_ |
| --- | --- | --- | --- | --- |
|  |  | mg L^-1^ |  |  |
| Susceptible | **2015** | 2.50 ±0.12 | 1.89 ± 0.16 |  |
|  | 2016 | 2.89 ± 0.25 | 1.68 ± 0.22 |  |
|  | 2017 | 2.84 ± 0.23 | 1.79 ± 0.23 |  |
| North |  |  |  |  |
| HaiPhong | 2015-1 | 22.33 ± 4.61 | 1.05 ± 0.22 | 8 |
|  | 2015-2 | 21.68 ± 2.87 | 1.45 ± 0.28 | 8 |
|  | 2016-1 | 26.88 ± 7.15 | 1.04 ± 0.36 | 10 |
|  | 2016-2 | 40.88 ± 4.49 | 2.71 ± 1.01 | 15 |
|  | 2017-1 | 20.13 ± 3.71 | 1.14 ± 0.23 | 7 |
|  | 2017-2 | 23.37 ± 3.53 | 1.29 ± 0.23 | 9 |
| NamDinh | 2015-1 | 21.12 ± 3.96 | 1.06 ± 0.20 | 8 |
|  | 2015-2 | 21.93 ± 2.87 | 1.36 ± 0.25 | 8 |
|  | 2016-1 | 29.77 ± 7.23 | 1.16 ± 0.41 | 11 |
|  | 2016-2 | 42.26 ± 4.67 | 3.23 ± 1.06 | 15 |
|  | 2017-1 | 21.23 ± 3.88 | 1.13 ± 0.22 | 8 |
|  | 2017-2 | 24.73 ± 4.17 | 1.29 ± 0.26 | 9 |
| VinhPhuc | 2015-1 | 19.11 ± 3.24 | 1.16 ± 0.21 | 7 |
|  | 2015-2 | 21.37 ± 3.60 | 1.21 ± 0.24 | 8 |
|  | 2016-1 | 24.63 ± 7.13 | 0.97 ± 0.34 | 9 |
|  | 2016-2 | 40.56 ± 5.69 | 2.40 ± 1.27 | 15 |
|  | 2017-1 | 20.13 ± 3.24 | 1.14 ± 0.20 | 7 |
|  | 2017-2 | 22.56 ± 3.74 | 1.20 ± 0.23 | 8 |
| Central |  |  |  |  |
| Hue | 2015-1 | 39.49 ± 5.38 | 3.36 ± 1.86 | 14 |
|  | 2015-2 | 39.91 ± 6.52 | 4.05 ± 2.44 | 15 |
|  | 2016-1 | 35.41 ± 8.62 | 1.57 ± 0.89 | 13 |
|  | 2016-2 | 40.62 ± 4.93 | 2.44 ± 1.08 | 15 |
|  | 2017-1 | 24.53 ± 4.07 | 1.26 ± 0.26 | 9 |
|  | 2017-2 | 27.88 ± 4.95 | 1.32 ± 0.30 | 10 |
| NgheAn | 2015-1 | 39.75 ± 4.87 | 3.12 ± 1.63 | 15 |
|  | 2015-2 | 39.52 ± 4.98 | 3.34 ± 1.73 | 14 |
|  | 2016-1 | 28.60 ± 6.20 | 1.06 ± 0.31 | 10 |
|  | 2016-2 | 36.57 ± 8.27 | 1.75 ± 0.98 | 13 |
|  | 2017-1 | 26.34 ± 3.74 | 1.42 ± 0.27 | 10 |
|  | 2017-2 | 26.22 ± 4.46 | 1.30 ± 0.27 | 10 |
| PhuYen | 2015-1 | 40.70 ± 4.28 | 3.07 ± 1.00 | 15 |
|  | 2015-2 | 42.25 ± 4.26 | 3.23 ± 0.96 | 15 |
|  | 2016-1 | 40.88 ± 5.55 | 2.86 ± 1.29 | 15 |
|  | 2017-1 | 26.19 ± 4.04 | 1.40 ± 0.29 | 10 |
|  | 2017-2 | 28.39 ± 4.85 | 1.24 ± 0.27 | 10 |
| South |  |  |  |  |
| AnGiang | 2015-1 | 42.26 ± 4.93 | 3.23 ± 1.12 | 15 |
|  | 2015-2 | 43.43 ± 4.48 | 3.63 ± 0.99 | 16 |
|  | 2016-1 | 42.07 ± 4.90 | 3.34 ± 1.13 | 15 |
|  | 2016-2 | 44.88 ± 6.30 | 3.91 ± 1.33 | 16 |
|  | 2017-1 | 31.85 ± 5.03 | 1.53 ± 0.40 | 12 |
|  | 2017-2 | 31.85 ± 4.81 | 1.53 ± 0.38 | 12 |
| LongAn | 2015-1 | 38.17 ± 2.95 | 3.14 ± 1.04 | 14 |
|  | 2015-2 | 39.15 ± 4.42 | 3.58 ± 1.62 | 14 |
|  | 2016-1 | 38.50 ± 5.52 | 2.16 ± 1.03 | 14 |
|  | 2016-2 | 42.25 ± 3.96 | 3.23 ± 0.90 | 15 |
|  | 2017-1 | 26.11 ± 3.92 | 1.52 ± 0.33 | 10 |
|  | 2017-2 | 30.07 ± 4.62 | 1.36 ± 0.30 | 11 |
| SocTrang | 2015-1 | 39.15 ± 3.84 | 3.58 ± 1.41 | 14 |
|  | 2015-2 | 42.25 ± 4.26 | 3.23 ± 0.96 | 15 |
|  | 2016-1 | 40.88 ± 4.59 | 2.69 ± 1.04 | 15 |
|  | 2016-2 | 42.07 ± 5.43 | 3.35 ± 1.25 | 15 |
|  | 2017-1 | 29.85 ± 4.63 | 1.47 ± 0.35 | 11 |
|  | 2017-2 | 29.82 ± 4.09 | 1.47 ± 0.31 | 11 |
