# Supplementary material for: Variation in susceptibility of eight insecticides in the brown planthopper Nilaparvata lugens in three regions of Vietnam 2015-2017
Source: PLoS One. 2018 Oct 5;13(10):e0204962. doi: 10.1371/journal.pone.0204962 (PMC6173402; doi:10.1371/journal.pone.0204962)
Supplement: S6 Table — RI50 were calculated by dividing LC50 with AVG LC50 (11.33) of the susceptible population. Year-1 and year-2 signify summer-autumn and winter-spring sampling of BPH. (DOCX) [file pone.0204962.s006.docx]

**S6 Table. Results of the bioassay with imidacloprid of BPH populations from North, Central and South Vietnam.** RI_50_ were calculated by dividing LC_50_ with AVG LC_50_ (11.33) of the susceptible population. Year-1 and year-2 signify summer-autumn and winter-spring sampling of BPH.

| Locality | Year-Season | LC_50_ ± SE | Slope ± SE | RI_50_ |
| --- | --- | --- | --- | --- |
|  |  | mg L^-1^ |  |  |
| Susceptible | 2015 | 10.87 ± 0.72 | 2.19 ± 0.36 |  |
|  | 2016 | 11.56 ± 0.84 | 1.69 ± 0.19 |  |
|  | 2017 | 11.56 ± 0.84 | 1.69 ± 0.19 |  |
| North |  |  |  |  |
| HaiPhong | 2015-1 | 64.67 ± 16.96 | 0.90 ± 0.21 | 6 |
|  | 2015-2 | 63.41 ± 11.77 | 0.92 ± 0.15 | 6 |
|  | 2016-1 | 92.78 ± 22.16 | 1.11 ± 0.36 | 8 |
|  | 2016-2 | 28.72 ± 7.86 | 0.68 ± 0.12 | 3 |
|  | 2017-1 | 68.43 ± 17.35 | 0.89 ± 0.20 | 6 |
|  | 2017-2 | 68.43 ± 16.87 | 0.89 ± 0.19 | 6 |
| NamDinh | 2015-1 | 68.41 ± 19.13 | 0.89 ± 0.22 | 6 |
|  | 2015-2 | 63.80 ± 11.66 | 0.97 ± 0.17 | 6 |
|  | 2016-1 | 97.82 ± 21.17 | 1.26 ± 0.40 | 9 |
|  | 2016-2 | 32.13 ± 8.89 | 0.66 ± 0.12 | 3 |
|  | 2017-1 | 68.42 ± 18.26 | 0.89 ± 0.21 | 6 |
|  | 2017-2 | 68.43 ± 16.87 | 0.89 ± 0.19 | 6 |
| VinhPhuc | 2015-1 | 61.20 ± 14.44 | 0.91 ± 0.19 | 5 |
|  | 2015-2 | 61.66 ± 12.50 | 0.96 ± 0.18 | 5 |
|  | 2016-1 | 81.27 ± 21.34 | 1.06 ± 0.34 | 7 |
|  | 2016-2 | 24.77 ± 5.77 | 0.67 ± 0.10 | 2 |
|  | 2017-1 | 60.89 ± 16.24 | 0.87 ± 0.19 | 5 |
|  | 2017-2 | 73.86 ± 20.13 | 0.91 ± 0.23 | 7 |
| Central |  |  |  |  |
| Hue | 2015-1 | 113.95 ± 15.84 | 2.40 ± 1.46 | 10 |
|  | 2015-2 | 123.13 ± 19.39 | 3.89 ± 3.18 | 11 |
|  | 2016-1 | 127.81 ± 16.31 | 2.69 ± 1.64 | 11 |
|  | 2016-2 | 127.82 ± 14.02 | 3.15 ± 1.35 | 11 |
|  | 2017-1 | 68.42 ± 19.14 | 0.89 ± 0.22 | 6 |
|  | 2017-2 | 125.09 ± 17.80 | 3.33 ± 2.86 | 11 |
| NgheAn | 2015-1 | 102.38 ± 20.82 | 1.68 ± 0.66 | 9 |
|  | 2015-2 | 120.46 ± 17.05 | 2.79 ± 2.66 | 11 |
|  | 2016-1 | 103.72 ± 26.99 | 1.40 ± 0.65 | 9 |
|  | 2016-2 | 121.64 ± 17.53 | 2.39 ± 1.42 | 11 |
|  | 2017-1 | 64.31 ± 18.96 | 0.82 ± 0.19 | 6 |
|  | 2017-2 | 83.50 ± 22.73 | 0.97 ± 0.28 | 7 |
| PhuYen | 2015-1 | 116.18 ± 10.46 | 2.99 ± 1.59 | 10 |
|  | 2015-2 | 131.75 ± 19.68 | 3.49 ± 1.84 | 12 |
|  | 2016-1 | 121.64 ± 14.18 | 2.39 ± 1.15 | 11 |
|  | 2017-1 | 72.47 ± 19.12 | 0.88 ± 0.21 | 6 |
|  | 2017-2 | 89.66 ± 23.74 | 1.06 ± 0.35 | 8 |
| South |  |  |  |  |
| AnGiang | 2015-1 | 126.57 ± 45.43 | 5.87 ± 9.00 | 11 |
|  | 2015-2 | 139.16 ± 14.47 | 4.14 ± 1.24 | 12 |
|  | 2016-1 | 135.54 ± 15.13 | 3.80 ± 1.35 | 12 |
|  | 2016-2 | 146.80 ± 15.43 | 3.48 ± 0.91 | 13 |
|  | 2017-1 | 75.15 ± 18.46 | 0.89 ± 0.20 | 7 |
|  | 2017-2 | 100.11 ± 19.60 | 1.33 ± 0.38 | 9 |
| LongAn | 2015-1 | 123.07 ± 16.39 | 3.90 ± 2.69 | 11 |
|  | 2015-2 | 122.55 ± 18.80 | 4.94 ± 3.77 | 11 |
|  | 2016-1 | 139.16 ± 19.77 | 4.14 ± 1.70 | 12 |
|  | 2016-2 | 143.60 ± 18.07 | 4.41 ± 1.46 | 13 |
|  | 2017-1 | 72.44 ± 19.59 | 0.88 ± 0.21 | 6 |
|  | 2017-2 | 92.24 ± 17.72 | 1.37 ± 0.36 | 8 |
| SocTrang | 2015-1 | 126.30 ± 36.21 | 5.06 ± 6.33 | 11 |
|  | 2015-2 | 135.09 ± 13.12 | 3.87 ± 1.19 | 12 |
|  | 2016-1 | 131.74 ± 13.53 | 3.49 ± 1.27 | 12 |
|  | 2016-2 | 139.16 ± 17.84 | 4.14 ± 1.53 | 12 |
|  | 2017-1 | 75.14 ± 19.23 | 0.84 ± 0.19 | 7 |
|  | 2017-2 | 99.51 ± 21.34 | 1.44 ± 0.49 | 9 |
