# Supplementary material for: Variation in susceptibility of eight insecticides in the brown planthopper Nilaparvata lugens in three regions of Vietnam 2015-2017
Source: PLoS One. 2018 Oct 5;13(10):e0204962. doi: 10.1371/journal.pone.0204962 (PMC6173402; doi:10.1371/journal.pone.0204962)
Supplement: S7 Table — RI50 were calculated by dividing LC50 with AVG LC50 (1.90) of the susceptible population. Year-1 and year-2 signify summer-autumn and winter-spring sampling of BPH. (DOCX) [file pone.0204962.s007.docx]

**S7 Table. Results of the bioassay with nitenpyram of BPH populations from North, Central and South Vietnam.** RI_50_ were calculated by dividing LC_50_ with AVG LC_50_ (1.90) of the susceptible population. Year-1 and year-2 signify summer-autumn and winter-spring sampling of BPH.

| Locality | Year-Season | LC_50_ ± SE | Slope ± SE | RI_50_ |
| --- | --- | --- | --- | --- |
|  |  | mg L^-1^ |  |  |
| Susceptible | **2015** | 1.91 ± 0.16 | 1.42 ± 0.14 |  |
|  | 2016 | 1.88 ± 0.23 | 1.37 ± 0.19 |  |
|  | 2017 | 1.91 ± 0.16 | 1.42 ± 0.14 |  |
| North |  |  |  |  |
| HaiPhong | 2015-1 | 14.63 ± 3.06 | 0.83 ± 0.13 | 8 |
|  | 2015-2 | 16.31 ± 4.11 | 0.80 ± 0.15 | 9 |
|  | 2016-1 | 25.97 ± 5.53 | 1.14 ± 0.30 | 14 |
|  | 2016-2 | 14.01 ± 2.39 | 1.00 ± 0.15 | 7 |
|  | 2017-1 | 13.89 ± 2.62 | 0.85 ± 0.12 | 7 |
|  | 2017-2 | 13.07 ± 2.37 | 0.84 ± 0.11 | 7 |
| NamDinh | 2015-1 | 15.10 ± 3.04 | 0.80 ± 0.12 | 8 |
|  | 2015-2 | 17.34 ± 4.25 | 0.82 ± 0.16 | 9 |
|  | 2016-1 | 27.20 ± 4.73 | 1.35 ± 0.32 | 14 |
|  | 2016-2 | 15.62 ± 3.03 | 1.00 ± 0.18 | 8 |
|  | 2017-1 | 15.55 ± 3.18 | 0.85 ± 0.14 | 8 |
|  | 2017-2 | 14.53 ± 3.25 | 0.80 ± 0.13 | 8 |
| VinhPhuc | 2015-1 | 12.57 ± 2.49 | 0.89 ± 0.14 | 7 |
|  | 2015-2 | 15.97 ± 3.79 | 0.79 ± 0.14 | 8 |
|  | 2016-1 | 23.43 ± 5.51 | 1.10 ± 0.32 | 12 |
|  | 2016-2 | 12.69 ± 2.02 | 1.01 ± 0.15 | 7 |
|  | 2017-1 | 12.57 ± 2.38 | 0.89 ± 0.13 | 7 |
|  | 2017-2 | 11.83 ± 2.13 | 0.88 ± 0.12 | 6 |
| Central |  |  |  |  |
| Hue | 2015-1 | 16.25 ± 2.99 | 1.10 ± 0.21 | 9 |
|  | 2015-2 | 17.45 ± 4.77 | 0.94 ± 0.25 | 9 |
|  | 2016-1 | 12.86 ± 2.92 | 0.84 ± 0.15 | 7 |
|  | 2016-2 | 16.76 ± 3.48 | 1.15 ± 0.26 | 9 |
|  | 2017-1 | 15.44 ± 3.76 | 0.82 ± 0.15 | 8 |
|  | 2017-2 | 16.31 ± 4.11 | 0.80 ± 0.15 | 9 |
| NgheAn | 2015-1 | 14.03 ± 2.01 | 0.98 ± 0.13 | 7 |
|  | 2015-2 | 16.19 ± 3.94 | 0.86 ± 0.18 | 9 |
|  | 2016-1 | 10.54 ± 2.11 | 0.77 ± 0.11 | 6 |
|  | 2016-2 | 14.10 ± 2.37 | 0.91 ± 0.13 | 7 |
|  | 2017-1 | 14.53 ± 3.25 | 0.80 ± 0.13 | 8 |
|  | 2017-2 | 14.63 ± 2.93 | 0.83 ± 0.13 | 8 |
| PhuYen | 2015-1 | 20.10 ± 2.71 | 1.29 ± 0.22 | 11 |
|  | 2015-2 | 21.22 ± 4.26 | 1.02 ± 0.23 | 11 |
|  | 2016-1 | 14.83 ± 2.81 | 0.97 ± 0.16 | 8 |
|  | 2017-1 | 16.41 ± 3.50 | 0.83 ± 0.14 | 9 |
|  | 2017-2 | 17.34 ± 4.12 | 0.82 ± 0.15 | 9 |
| South |  |  |  |  |
| AnGiang | 2015-1 | 18.77 ± 3.34 | 1.19 ± 0.22 | 10 |
|  | 2015-2 | 18.38 ± 3.14 | 1.00 ± 0.16 | 10 |
|  | 2016-1 | 16.11 ± 2.84 | 0.98 ± 0.16 | 8 |
|  | 2016-2 | 17.96 ± 4.32 | 1.01 ± 0.23 | 9 |
|  | 2017-1 | 17.34 ± 4.38 | 0.82 ± 0.16 | 9 |
|  | 2017-2 | 18.36 ± 4.89 | 0.81 ± 0.17 | 10 |
| LongAn | 2015-1 | 16.43 ± 2.99 | 1.13 ± 0.20 | 9 |
|  | 2015-2 | 16.22 ± 2.68 | 1.05 ± 0.17 | 9 |
|  | 2016-1 | 13.20 ± 3.48 | 0.75 ± 0.14 | 7 |
|  | 2016-2 | 14.10 ± 2.07 | 0.91 ± 0.11 | 7 |
|  | 2017-1 | 15.35 ± 3.83 | 0.79 ± 0.14 | 8 |
|  | 2017-2 | 14.76 ± 2.99 | 0.86 ± 0.14 | 8 |
| SocTrang | 2015-1 | 17.20 ± 2.30 | 1.14 ± 0.16 | 9 |
|  | 2015-2 | 18.57 ± 3.51 | 1.24 ± 0.29 | 10 |
|  | 2016-1 | 14.10 ± 2.50 | 0.91 ± 0.14 | 7 |
|  | 2016-2 | 17.96 ± 4.32 | 1.01 ± 0.23 | 9 |
|  | 2017-1 | 16.31 ± 3.72 | 0.80 ± 0.14 | 9 |
|  | 2017-2 | 17.34 ± 4.38 | 0.82 ± 0.16 | 9 |
