# Supplementary material for: Variation in susceptibility of eight insecticides in the brown planthopper Nilaparvata lugens in three regions of Vietnam 2015-2017
Source: PLoS One. 2018 Oct 5;13(10):e0204962. doi: 10.1371/journal.pone.0204962 (PMC6173402; doi:10.1371/journal.pone.0204962)
Supplement: S8 Table — RI50 were calculated by dividing LC50 with AVG LC50 (26.34) of the susceptible population. Year-1 and year-2 signify summer-autumn and winter-spring sampling of BPH. (DOCX) [file pone.0204962.s008.docx]

**S8 Table. Results of the bioassay with pymetrozine of BPH populations from North, Central and South Vietnam.** RI_50_ were calculated by dividing LC_50_ with AVG LC_50_ (26.34) of the susceptible population. Year-1 and year-2 signify summer-autumn and winter-spring sampling of BPH.

| Locality | Year-Season | LC_50_± SE | Slope± SE | RI_50_ |
| --- | --- | --- | --- | --- |
|  |  | mg L^-1^ |  |  |
| Susceptible | 2015 | 27.33 ± 4.38 | 1.47 ± 0.30 |  |
|  | 2016 | 26.78 ± 4.21 | 1.52 ± 0.33 |  |
|  | 2017 | 24.90 ± 4.16 | 1.36 ± 0.27 |  |
| North |  |  |  |  |
| HaiPhong | 2015-1 | 395.31 ± 50.95 | 3.33 ± 1.77 | 15 |
|  | 2015-2 | 398.00 ± 46.81 | 3.06 ± 1.63 | 15 |
|  | 2016-1 | 262.20 ± 56.23 | 0.98 ± 0.27 | 10 |
|  | 2016-2 | 391.70 ± 56.06 | 3.58 ± 2.05 | 15 |
|  | 2017-1 | 342.57 ± 97.02 | 1.59 ± 1.16 | 13 |
|  | 2017-2 | 391.57 ± 56.17 | 3.58 ± 2.05 | 15 |
| NamDinh | 2015-1 | 395.29 ± 50.98 | 3.34 ± 1.77 | 15 |
|  | 2015-2 | 397.92 ± 46.90 | 3.07 ± 1.62 | 15 |
|  | 2016-1 | 254.17 ± 52.15 | 0.98 ± 0.23 | 10 |
|  | 2016-2 | 434.27 ± 68.32 | 3.63 ± 1.52 | 17 |
|  | 2017-1 | 403.11 ± 67.12 | 3.85 ± 2.37 | 15 |
|  | 2017-2 | 399.04 ± 74.05 | 4.05 ± 2.77 | 15 |
| VinhPhuc | 2015-1 | 382.10 ± 44.77 | 3.09 ± 1.58 | 15 |
|  | 2015-2 | 383.04 ± 43.60 | 2.72 ± 1.46 | 15 |
|  | 2016-1 | 236.15 ± 51.84 | 0.85 ± 0.19 | 9 |
|  | 2016-2 | 454.36 ± 59.97 | 2.41 ± 0.75 | 17 |
|  | 2017-1 | 398.03 ± 46.83 | 3.06 ± 1.63 | 15 |
|  | 2017-2 | 387.52 ± 62.63 | 3.81 ± 2.48 | 15 |
| Central |  |  |  |  |
| Hue | 2015-1 | 236.05 ± 34.91 | 1.76 ± 0.41 | 9 |
|  | 2015-2 | 330.16 ± 61.68 | 1.64 ± 0.64 | 13 |
|  | 2016-1 | 305.39 ± 55.06 | 1.36 ± 0.39 | 12 |
|  | 2016-2 | 371.33 ± 48.1 | 2.23 ± 0.84 | 14 |
|  | 2017-1 | 395.42 ± 50.97 | 3.32 ± 1.79 | 15 |
|  | 2017-2 | 395.27 ± 51.00 | 3.34 ± 1.77 | 15 |
| NgheAn | 2015-1 | 222.11 ± 33.00 | 1.74 ± 0.41 | 8 |
|  | 2015-2 | 310.69 ± 53.90 | 1.58 ± 0.49 | 12 |
|  | 2016-1 | 283.66 ± 51.80 | 1.28 ± 0.35 | 11 |
|  | 2016-2 | 314.48 ± 49.36 | 1.66 ± 0.46 | 12 |
|  | 2017-1 | 246.85 ± 53.06 | 0.92 ± 0.22 | 9 |
|  | 2017-2 | 383.09 ± 43.30 | 2.78 ± 1.41 | 15 |
| PhuYen | 2015-1 | 268.74 ± 35.68 | 2.12 ± 0.6 | 10 |
|  | 2015-2 | 357.2 ± 56.02 | 1.85 ± 0.7 | 14 |
|  | 2016-1 | 313.59 ± 49.67 | 1.64 ± 0.46 | 12 |
|  | 2017-1 | 395.41 ± 50.82 | 3.33 ± 1.76 | 15 |
|  | 2017-2 | 403.07 ± 66.93 | 3.85 ± 2.36 | 15 |
| South |  |  |  |  |
| AnGiang | 2015-1 | 337.69 ± 49.15 | 2.03 ± 0.71 | 13 |
|  | 2015-2 | 372.04 ± 47.36 | 2.27 ± 0.84 | 14 |
|  | 2016-1 | 448.78 ± 78.09 | 3.91 ± 1.65 | 17 |
|  | 2016-2 | 432.41 ± 70.97 | 3.71 ± 1.6 | 17 |
|  | 2017-1 | 406.48 ± 61.51 | 3.67 ± 2.1 | 16 |
|  | 2017-2 | 403.07 ± 66.94 | 3.85 ± 2.36 | 15 |
| LongAn | 2015-1 | 352.44 ± 39.36 | 2.66 ± 1.18 | 13 |
|  | 2015-2 | 327.17 ± 54.59 | 1.79 ± 0.67 | 13 |
|  | 2016-1 | 434.32 ± 68.23 | 3.63 ± 1.51 | 17 |
|  | 2016-2 | 422.51 ± 57.12 | 3.23 ± 1.29 | 16 |
|  | 2017-1 | 406.45 ± 61.44 | 3.67 ± 2.09 | 16 |
|  | 2017-2 | 395.25 ± 51.02 | 3.34 ± 1.78 | 15 |
| SocTrang | 2015-1 | 348.31 ± 43.41 | 2.36 ± 0.93 | 13 |
|  | 2015-2 | 346.45 ± 45.56 | 2.26 ± 0.93 | 13 |
|  | 2016-1 | 420.64 ± 59.63 | 3.35 ± 1.37 | 16 |
|  | 2016-2 | 422.67 ± 57.02 | 3.22 ± 1.29 | 16 |
|  | 2017-1 | 397.87 ± 46.95 | 3.07 ± 1.62 | 15 |
|  | 2017-2 | 353.09 ± 58.33 | 1.77 ± 0.69 | 13 |
